# A Treatise on Tetanus, Being an Essay for Which the Jacksonian Prize Was Awarded by the College of Physicians

**Published:** 1837-01-01

**Authors:** 


A Treatise on Tetanus, being the Essay for which the Jacksonian Prize, for the Year 1834, was awarded by the Royal College of Surgeons, in London. By *Thomas Blizard Curling*, Assistant Surgeon to the London Hospital, and Lecturer on Morbid Anatomy. 8vo. pp. 236. London, 1836.

Our readers may feel surprized at observing in our pages a Review of a new work on Tetanus. They may experience some difficulty in reconciling the phenomenon with our professed creed of utilitarianism.

But softly gentlemen. We will soon convince you that we are not straying from the spirit, nor indeed from the letter of our professions, and this review will be speedily acknowledged to be a very proper and a very useful one.

The book before us is not what might be expected—an elaborate disquisition on the cause of the disease—or a bold annunciation of a cure for it. It does not say that tetanus consists essentially in redness here or redness there—in inflammation of the spinal marrow, or in a colon full of fæces—nor does it assert, that the world is under a delusion with respect to the incurability of the disorder, and that Mr. Blizard Curling experiences no difficulty in subduing it.

It is in fact a little work conceived and executed in the right spirit of philosophy. The author, knowing how fallacious the experience of any one man in such a malady must be, has gone the proper way to work, and collecting and comparing a large number of published cases, he has taken a statistical survey of their features. This is just what is wanted in such a complaint. No method is more calculated to dispel prejudice, or to enlarge our views with respect both to the causes, the march, and the result of a disease of this description. Let Mr. Curling say what he has done.

"In order to acquire more accurate knowledge respecting this disease, as well as to obtain data to reason from, 128 cases of traumatic Tetanus have been carefully examined, analysed, and arranged in a table, distinguishing the sexes, and giving the ages of the patients, the nature of the injury, the time subsequent to the infliction of the wound at which the disease first showed itself, the result, and at what time after the appearance of symptoms, a general view of the treatment, and the sources from which the cases have been obtained. It would have been desirable to have added to these particulars an account of the trade or occupation of each patient, and of the state of the pulse; but as in most instances nothing is said in respect to these points, it is obvious that the columns devoted to them must have been exceedingly imperfect." *Introd.* ix.

The false facts of medicine have been often denounced. They are infamous enough. But the half facts are equally dangerous. Not to tell the whole truth is, in some cases, virtually to tell a lie. This system of constructive mendacity prevails to no trifling degree. A man has no inducement to publish his failures. Why should he intrude his want of success upon the world? But he has a very strong inducement to trumpet his success; accordingly he does so. If such a man has a fatal case of tetanus, there is no necessity for his relating that. There have been many other fatal cases of tetanus, and a fresh one is not likely to be very instructive. But if he happens to have a case which does well, of course he would not keep that back, and, in order that it may be more impressive, nothing is said about those which have done ill. The consequence is, that if we trusted books we should believe that tetanus is a very curable disease, which those of us who have a pennyworth of experience know that it is not. In Mr. Curling's work, for instance, one hundred and twenty-eight examples have been taken indiscriminately from various sources; *and although it is well known that by far the greater proportion of the subjects of this disease never recover, yet fifty-eight, or nearly one-half of them, are instances of cure.* This is pretty well, and speaks highly for our professional literature. It almost justifies the contempt which some express for its recorded facts.

Of course we have no intention of presenting a connected account of the complaint. All we intend doing, is selecting some particulars which have been illustrated, confirmed, or corrected by the comparison of these numerous data. This is the only plan adapted for us, and it must necessarily give the article a disjointed character. But that is of no moment, and we must proceed to business.

The work consists of four chapters and an appendix. The chapters are devoted successively to—a general account of tetanus—its pathology—its treatment—and to tetanus nascentium; the Appendix is composed of the table of cases already alluded to, and of their bibliography.

### 1. GENERAL ACCOUNT OF TETANUS.

The first point to which we may allude, is the important practical division of tetanus into the acute and chronic, the former very fatal, the latter comparatively trivial. This distinction has been frequently insisted on. Yet in societies and in books we find surgeons overlooking it, and recording the cure of chronic tetanus with as much formality as if it were not common enough.

Passing over the symptoms of tetanus, unfortunately too familiar, we may pause to survey the peculiar characters that it imprints upon the countenance.

The expression of this, although sufficiently uniform to be characteristic of the disease, is not invariably the same in every case. Aretæus observes, that distortion of the face is so great that patients are not recognized by their most intimate friends. A case of idiopathic tetanus is related by Dr. W. Farr, in the fourth volume of the Medical Observations and Inquiries, in which the spasms of the muscles of the face had so deeply impressed the marks of age on the countenance of the patient, who was only twenty-six years of age, as to induce all who saw him in that state to believe that he was at least sixty. Mr. Curling once observed the same circumstance in an equally remarkable degree.

Tetanus, we all know, is a horribly painful disease. The patient usually suffers agony during the continuance of the spasms. But Sir Gilbert Blane mentions, on the authority of a surgeon in the navy, an extraordinary case of Tetanus which proved fatal in four days, where the sensation, excited by the violent muscular contractions, was a sort of tingling of a pleasurable nature. In case 81 in the table, no uneasiness was occasioned by the spasms of the muscles of the jaw and throat. Pain at the præcordium, dependent, probably, on spasm of the diaphragm, has been called the pathognomonic symptom of tetanus, and some writers have asserted that, when it occurs, the disease invariably proves fatal. The cases collected by our author prove that this is not the fact, and, in many instances, the patients recovered, although this symptom was severe.

Mr. Curling has generally found the pupil contracted in tetanus.

The spasms, it is notorious, are essentially seated in the muscles supplied with nervous influence from the brain and spinal-marrow. Yet patients sometimes die so suddenly that implication of the heart has been accused of giving rise to the occurrence. Dr. C. H. Parry has attached great importance to the state of the heart, which, he imagines, becomes exhausted, and first loses its vital powers. Dr. Currie remarks that " the patient, where the case terminates fatally, probably dies at last from spasmodic affection extending to the heart." Mr. Travers is likewise of opinion that the disease travels ultimately to the heart and other viscera, a view which has been advocated by Mr. Howship, who has published a case, where the patient, in describing his sufferings, is reported to have said, that " he felt that his heart was at times ready to break with the distress of its pain." This organ, when examined eleven hours after death, the body being still warm, was found so much reduced in bulk as not to occupy a fourth part of the cavity of the pericardium, appearing to be very unnaturally shortened from the basis to the apex. The resistance upon pressing the muscular ventricles resembled that of a very firm and even horny substance rather than of a mere fleshy cavity. The substance of the heart felt in every part unnaturally firm, to a degree far beyond what can be well conceived. The auricles, as well as the ventricles, were greatly contracted. The sides of the left ventricle were found in the closest contact, so much so that when a section of the heart was made, the exact situation of the cavity was not immediately to be perceived. About half an ounce of blood still remained in the right ventricle. The auricle on the right side contained about an ounce of blood, that on the left a much less proportion. The heart was removed from the body, and upon being examined a few hours afterwards, it was found to have become completely relaxed, was much larger and softer, as well as very flaccid, having entirely lost that very peculiar and remarkable state of tone which it possessed at the time of its being first inspected. No other morbid appearance is described, and Mr. Howship concludes "that it was on every side clear, that the muscular structure of the heart had in this case become at length subject to the same affection which, during the earlier stages of the disease, was confined principally to those parts of the body subservient to voluntary motion." Mr. Curling justly remarks that the correctness of this conclusion is by no means certain. For, in the first place, the heart may often be found extremely firm after death—and, secondly, evidence is against this being the usual condition of the organ in tetanus. Dr. Reid, for example, after relating the morbid appearances in a case of Tetanus, observes, that "the difference of the ganglionic system from the spinal was well marked, by the influence of the disease not extending in a similar manner to the muscular organs of both systems. This was marked by the muscular parts of the heart and intestines being paler than natural, while the muscular organs belonging to the spinal system were remarkably florid and rigid." During the continuance of the complaint, the heart displays no symptom of being affected with the spasms that convulse the voluntary muscles, on the contrary, its action is generally accelerated. Of course it is impossible to affirm that the heart is in no case affected, but, in the great majority, there is no reason to believe that it is so, and, even where it has been supposed to be concerned, a simpler explanation of the sudden death may be offered.

"Dr. Parry has, however, gone further, and has attempted to establish grounds for forming a probable conclusion as to the event of the disease by attending to the state of the pulse. He says, 'If in an adult the pulse, by the fourth or fifth day, does not reach one hundred or perhaps one hundred and ten beats in a minute, I believe the patient almost always recovers. If, on the other hand, the pulse on the fourth day is one hundred and twenty or more in a minute, few instances will, I apprehend, be found in which he will not die.' The accuracy of a prognosis founded upon the state of the heart's action, was verified in six instances. I regret that the state of the pulse having been so rarely recorded in the reports of the cases composing the table, it is impossible to obtain any satisfactory data upon this point; but Dr. Parry's rule is not sustained by the testimony of other writers of experience. Dr. Morrison, who had ample opportunities for observation in Demerara, states that the pulse seldom exceeds eighty. In a boy, who fell a victim to the disease on the third day, in the absence of spasms the pulse was ninety-eight, but he has never remarked it higher than this. Dr. Hennen found the pulse in the cases that he had met with, astonishingly unaffected, and the same was observed by Sir James Macgregor. In cases 19, 33, 36, 55, 57, 60, 87, 90, 95, 115, and 125, although the pulse exceeded one hundred and ten beats, the patients recovered. In all those cases which I have witnessed, the action of the heart, as well as the respiratory movements, has been in some degree accelerated; and during the paroxysms there has generally been a further increase of about ten or twelve pulsations in the minute, whilst towards the close of the disease the pulse has been feeble, irregular, and sometimes intermittent. In case 113 the pulse was as much as one hundred and eighty during the paroxysms. This increased action is readily accounted for. For in any violent exertion of the muscles, as in running, the blood being precipitated with increased rapidity to the right side of the heart, the force and velocity of the circulation will necessarily be increased, and as a consequence respiration must also become accelerated. I conceive, therefore, that in Tetanus the frequency of the pulse and the hurried respiration must be, in a great degree, ascribed to the forcible contractions to which the muscles are excited, especially during the paroxysms. Yet the danger of the disease is not exactly proportionate to the intensity of the spasms, but is influenced, as will be shown hereafter, by the particular class of muscles affected. It is necessary also to notice, in an inquiry into the state of the pulse, that many of the remedies which are so often exhibited in large doses, may exert considerable influence in increasing the heart's action, as camphor, mercury, opium, and various stimulants. So that viewing all these circumstances, the condition of the pulse must be regarded as a very fallacious test of the severity or acuteness of the disease, and cannot therefore be depended upon as a guide in forming an accurate prognosis, Dr. Parry's rule being, in fact, neither sustained by experience, nor based upon any rational theory." 16.

It has been imagined that even the middle coat of arteries has been affected with the spasm—an opinion extremely improbable in itself, and supported only by a case of Mr. Liston's, which really proves nothing whatever.

The costiveness attendant upon tetanus, has led some authors to conceive that the muscular fibres of the intestines are involved. But this conjecture is of very dubious propriety, and there are many reasons, which we need not now enter on, why costiveness should occur, independently of spasm of the muscular coat of the bowel.

Retention of urine is sometimes occasioned, as in case 10, probably by spasm of the compressor urethræ, and great difficulty has thereby been experienced in the attempt to introduce a catheter for the purpose of evacuating the bladder. Mr. Morgan has seen priapism in tetanus. Mr. Curling never witnessed it, nor have we.

The muscles are observed to be relaxed during sleep, a striking example of which occurred to Mr. Mayo, in a boy who recovered from the disease. On visiting his patient before the symptoms were subdued, Mr. Mayo found him asleep, and remarked that he lay perfectly relaxed. The abdominal muscles were soft and yielding, and had not the least tension. The boy was awakened, and at the instant the full tension of the muscles returned. Not being further disturbed he fell asleep in a few minutes, when the muscles again became relaxed, and again, on his being awakened, resumed the state of spasm. Mr. Curling has, in several instances, seen the same thing. The fact is, as Mr. Curling remarks, that during the height of the disease, it appears impossible for sleep to occur, and it is in chronic tetanus, or during recovery from the acute, that sleep is likely to ensue. If, however, from exhaustion, or from the influence of powerful narcotics, sleep is obtained, it can last only till re-action enough is established to support the renewal of spasmodic contractions.

In tetanus the cutaneous secretion is increased, and has often a pungent and peculiar smell. It is singular that Baron Larrey should attach so much importance to the occurrence of perspiration, which, on the chest and abdomen, he describes as critical. We say that it is singular, because we constantly see tetanic patients bathed in sweat, without the slightest amelioration taking place.

The skin usually feels hot, and it appears that the heat of the body is sometimes above the natural standard. M. Prévost, of Geneva, had a patient, a boy twelve years of age, affected with Tetanus, which was accompanied by an extraordinary development of heat. A thermometer, placed in the axilla, was raised to 110° 75 Fahr. Reckoning the original temperature at 98°, which is above the average for that age, there was an elevation of 12½° Fahr. In a case of traumatic Tetanus described by Dr. Bright, the temperature in the axilla on the third day was 105°. It is not surprising that the temperature should be raised. Violent muscular exercise, a quickened circulation from any cause, will do this. The actual mode in which the animal heat is generated, is so buried in obscurity, that conjectures on its alterations in a particular case are too often idle. In one case the condition of the circulating system appears to be its principal regulator. In another the nervous system plays as conspicuous and a more mysterious part. In a case of fracture of the spine, for example, which occurred at St. George's Hospital, the heat on the surface rose, we believe, to 110°. All we can do at present is, to watch and to record the facts.

*Mode of Termination.*

Tetanus may terminate in recovery, or it may not. When recovery does take place, it is very gradual. Of fifty-eight cases in the table, which terminated successfully, eight were cured in the course of a week; three in ten days; four in a fortnight; four recovered at the end of three weeks; fifteen at the end of a month; four after five weeks; eight after six weeks; three at the end of eight weeks; three after two months; and in two the symptoms were not removed till after three months. Even after the spasms have subsided, it is often a long time before the muscles can regain their tone and freedom of action. In cases 23 and 119, the muscles continued stiff for some length of time after recovery. In case 22, at the end of nine months after the patient first became affected, although enjoying good health, yet on catching cold he was still troubled with a stiffness about the jaws. In a case which Mr. Curling witnessed, rigidity of the muscles of the lower jaw continued for six months after the attack. In a case related by Dr. Currie, at the end of three years, it is stated that "his features retained the indelible impression of the disease." When we remember the soreness left by a bruise or a cramp, we cannot wonder at the effects of such violent contractions of the muscular fibre as occur in tetanus.

"The immediate cause of death from Tetanus is variously explained. The reasons for the opinion that it is attributable sometimes to a spasm seizing the heart have already been controverted. The ordinary causes are asphyxia, and exhaustion, and death is retarded or accelerated, according to the violence of the symptoms, the class of muscles principally affected, and the constitution of the patient. Asphyxia may be occasioned either by spasmodic closure of the glottis only, or by the spasms entirely suspending the regular action of the diaphragm, and of the other muscles concerned in increasing and diminishing the capacity of the thorax. When produced in the former way, death is somewhat sudden, occurring during a severe paroxysm, warning having perhaps been given by a sense of suffocation during a previous exacerbation. In the other case, death approaches slowly, the respiration being for several hours hurried, laborious, and irregular; the lungs become gradually congested, the motions of the chest being impeded, the expulsion of the inspired air is prevented, and thus a fatal termination tardily ensues. Often, however, the two causes acting together speedily put an end to the patient's sufferings. When death takes place from exhaustion, violent spasms, attended with profuse perspiration, have perhaps been prolonged through many days, or even weeks, the patient during that time having been unable to swallow nutriment. At length the paroxysms become less acute and frequent, the pulse flags, the muscles relax, the eyes become dim, and he gradually sinks, the vital powers being at length worn out by the severe and painfully protracted spasms." 26.

Tracheotomy has been recommended. Of course it could only be of use, where there is contraction of the glottis. When done it must be quickly done, and Mr. Curling does not know that it has ever actually been done. Probably it would be of little service, but as it might protract life and augment chances, it might, in proper cases, be resorted to.

*Statistics of Tetanus.*

"There are no data from which a correct estimate can be formed of the frequency of Tetanus in comparison with other diseases, of the probability of its occurrence after wounds, or of its mean rate of mortality, either in the traumatic form or in the idiopathic. Sir James Macgregor alludes to several hundred cases observed in the campaigns in Spain and Portugal, very few of which were cured. Mr. O'Beirne mentions, that out of about two hundred cases which he witnessed in the Peninsular war, not one recovered, and on a rough calculation it was generally considered to attack in the proportion of one in two hundred wounded. Tetanus must occur oftener amongst an equal number of wounded after a battle than in civil life, in consequence of the men being more exposed to the predisposing causes of the disease; on which account, and from the greater severity of the injuries, the majority of them being inflicted by fire-arms, the probability of recovery must also be much less. Mr. Howship has published the particulars of thirteen cases of Tetanus occurring after injuries of different severity, most of them being gun-shot wounds, of which ten were fatal. Sir Gilbert Blane mentions, that the number of wounded in the actions of April 1782 in the West Indies, was eight hundred and ten, of whom twenty, or one in forty and a half, were attacked with Tetanus, seventeen of which number died. Mr. Dickinson, surgeon at Grenada, met with thirteen cases of traumatic Tetanus; nine died and four were cured. He also witnessed ten cases of the idiopathic disease, of which six terminated fatally. In the table are included seventy fatal cases, from which it appears that the disease is equally fatal at every period of life, from the age of ten to forty-five." 29.

Idiopathic tetanus is well known not to be so fatal as the traumatic, but it is more so in hot climates than in temperate ones.

Tetanus is both less frequent and less fatal in the female sex than in the male. Of the one hundred and twenty-eight cases in the table, sixteen were females, being to the males in the proportion of one to eight. Of these sixteen cases four only were fatal, whereas, out of the one hundred and twelve cases of males, sixty-six, or more than one-half, died. Women are less exposed both to the predisposing and exciting causes of the disease. This would account for its infrequency in their sex. Its smaller rate of mortality, when it does occur, may possibly depend on their more delicate conformation, for the robust and muscular are most subject to it.

In most of the cases in the table, the patients were attacked between the ages of ten and fifty; four only having occurred after the fiftieth year, and three before the age of ten. The duration of the disease appears to be influenced by circumstances which we do not exactly understand. The extent of the original injury affects it. Sometimes it is very quickly fatal. Wepfer saw an infant, to whom tetanus proved fatal in three minutes; we may doubt if this was a pure instance of the disease. Another rapidly fatal case is mentioned by the late Professor Robison of Edinburgh. A negro having scratched his thumb with a piece of broken china, was seized with Tetanus, and in a quarter of an hour after this slight injury he was dead. In case 102, related in the Dictionnaire des Sciences Médicales, the disease proved fatal in twelve hours. In case 62, the patient died in twenty hours; in case 94, in twenty-six hours; and in cases 15, 53, and 96, in forty hours. Mr. Dickinson mentions an instance of a negro, twelve years of age, in which the disease ended fatally in twenty-two hours. In a case that occurred to Mr. Adams, of the London Hospital, the patient died in sixteen hours.

In the table, fifty-three cases were fatal within eight days after the appearance of symptoms; eleven by the following day; fifteen on the second day after, eight on the third, seven on the fourth, three on the fifth, four on the sixth, three on the seventh, and two on the eighth, but very few having lasted longer. In case forty-two, which is taken from Morgagni, the symptoms are related to have continued for upwards of twenty days before the patient was destroyed. The experience of Dr. Lionel Chalmers in acute idiopathic tetanus in South Carolina, accords very closely with the average duration of the traumatic disease, as estimated from the table. He states, that patients generally die in twenty-four, thirty-six, or forty-eight hours, and very rarely survive the third day, but when the disease is less acute, few are lost after the ninth or eleventh day.

### *Causes of Tetanus.*

When we say that traumatic tetanus has been seen to follow almost every kind of injury, almost all operations, even cupping, the drawing of a tooth, or the injection of a hydrocele, we have given a summary of what is known on these matters. It is said to occur most frequently after punctures and lacerations of fascious structures and of nerves, and after wounds of the hands and feet. It appears from the table, that in thirty-four instances the disease was occasioned by injuries, many of them very trivial, of the hands and fingers; and in thirty-five cases by injuries to the feet or toes. In sixty-four instances, the wounds were on some part of the lower extremities, in forty-six, on the upper extremities. Mr. O'Beirne mentions, that he never witnessed the disease after an injury of the head, but in eleven cases in the table, the wounds were either on the face or scalp. Dr. Morrison notices three cases in negroes, where it originated from a severe flagellation. Baron Larrey has recorded an instance in which the disease was produced by a fish-bone lodged in the fauces. Mr. Morgan witnessed two cases, consequent upon the blows inflicted by a schoolmaster with his cane,—they both terminated fatally. Dr. Rush remarked that there was invariably an absence of inflammation in the wounds causing the disease. In many of the cases in the table, the wound was healed, and almost forgotten.

The interval between the infliction of the injury and the accession of tetanic symptoms varies considerably. In the case mentioned on the authority of Professor Robison, the disease appeared almost instantaneously. In case 114, only an hour elapsed. In case 102, symptoms were evinced within two hours after the injury; and in case 19, in eleven hours. In eighty-one instances, symptoms were first developed from the fourth to the fourteenth day after the wound; and in nineteen they were evinced on the tenth day. In thirteen cases, witnessed by Mr. Dickinson, at Grenada, the period varied from eight to fourteen days.

The longer the interval, the more chronic the disease, and, consequently, the greater the chance of recovery. In thirteen cases, symptoms of tetanus occurred about three weeks after the wound, but four only were fatal; and of seven cases, in which they did not make their appearance till after a month, only two ended fatally. In the returns made by Sir James Macgregor, the interval never exceeded three weeks, and in all the cases witnessed by Sir Benjamin Brodie, with one exception, the disease showed itself in the course of the second week after the reception of the wound. In that particular case, the symptoms were developed on the seventeenth day, but they were chronic, and the patient recovered. Sir Gilbert Blane, who had numerous opportunities of observing the disease in the navy, states that he has known it commence at all periods, from the second day to the end of the fourth week. Mr. Ward, of Manchester, saw it follow ten weeks after a burn. Was that traumatic tetanus?

Tetanus is more frequent in sultry than in temperate climates—prevails in hot seasons, or during sudden changes from heat to cold, especially in a moist state of atmosphere—and is apt to follow exposure to damp and cold. Dr. R. Huck, physician to the army in the last century, states that, of thirteen wounded men whom he had seen afflicted with lock-jaw, nine received their wounds at the attack of the French lines at Ticonderoga, in the year 1758, and remained exposed to the cold air, the night after the action, in open boats upon Lake George. M. François, of Auxerre, observes, that on board the Amazon frigate, before Charles Town, during the American war, after some stormy and very wet weather which had succeeded a continuance of dry, most of those wounded by fire-arms were attacked with Tetanus on the fourteenth day. Dr. Hennen states, that in almost all instances the patients had been exposed to a stream of air directly blowing upon them. He mentions, that many of the wounded were seized with it after the battle of El-Arich, and after the capture of Jaffa, when they were placed in tents on the wet ground exposed to constant rain. Baron Larrey noticed, that the disease was unfrequent during an equable temperature, but common when the wounded were exposed during the night to the immediate impression of cold and damp air. The cold bath, and drinking cold water when heated have brought it on, in short the efficiency of cold in producing both idiopathic and traumatic tetanus is established.

In a note we are informed of some striking facts with regard to the production of tetanus in the horse. Thus Hurtrel d'Arboval relates, that twenty-four horses were castrated on the same day at Bec, in the department de l'Eure. They were afterwards led four times in the day through a pond of water supplied from a very cold spring. Sixteen of them died between the tenth and fifteenth day after the operation. At Rennes, a horse after castration was exercised until he was covered with perspiration, and then suddenly plunged into the river. This was repeated three times, and the animal died tetanic. Tetanus is frequent in horses, occurs sometimes in lambs, and has been seen in parrots. It is rarely idiopathic in horses, and in them the traumatic is not so fatal as in man, the average of recoveries being rated by Mr. Sewel at three in five.

Tetanus is common in the West Indies, where the Negroes suffer more than the whites, probably because they are more exposed. The disease, however, is not so common in the islands as it was. That it is not so frequent in the navy will be evident, when we state that Dr. Lind, who was physician to the fleet, about the middle of the last century, has recorded, that after amputation, five cases out of six generally proved fatal from an attack of lock-jaw!

On the prognosis and diagnosis we need not dwell. With respect to the former it is enough to say that, even in the worst forms recoveries do take place, and that in chronic tetanus they are still more common.

### PATHOLOGY OF TETANUS.

This occupies the second chapter. Our author considers, first, the morbid appearances of Tetanus; secondly, the theory of the disease. The morbid appearances are considered under the heads: Lesions of the Nervous System—Lesions of the Muscular System—Lesions of the Viscera and other parts of the Body.

#### 1. *Lesions of the Nervous System.*

The first part of this system investigated is the brain, spinal cord, and their investing membranes. The changes of these structures are so dubious and so vague, that we shall not dwell much on them.

The principal alterations of the brain and its membranes found after death from Tetanus are:—congestion of the sinuses; the vessels of the pia-mater filled with florid blood; more or less increased vascularity of the cerebral substance; slight serous effusion between the membranes and in the ventricles.

"In some rare instances changes of a permanent nature have been observed. Bouillaud relates the case of a boy who had during life symptoms simulating Tetanus, in which a tubercle the size of a large egg, and five or six smaller ones, were found after death in the substance of the right hemisphere. In a fatal case of Tetanus, described by Dr. Bright in his Medical Reports, (case 39,) in which the symptoms appeared at the end of four weeks after a blow on the left side of the head, on examination after death, a collection of pus about the extent of a large nutmeg, was found in the substance of the brain about the middle of the middle lobe, close to the part where the blow had been received. This pus was contained in a firm cyst as thick as cartridge paper, exceedingly vascular, and almost made up of minute vessels: the cyst adhered to the dura mater, and seemed not improbably to be formed originally from a fold of the pia mater." 47.

When we turn to the medulla and its membranes, we find that serous effusion, with increased vascularity, is generally observed in the latter, and a turgid condition of the blood-vessels about the origin of the nerves. Yet, in many recorded cases, the spinal marrow and its coverings were natural. This was the fact in the examinations made by Sir Benjamin Brodie.

It is curious that, vague as these appearances are, incompetent as they must be to establish the existence of active inflammation, there have yet been many, especially abroad, to insist that inflammation of the brain, or spinal cord, or their membranes, is the essential cause of tetanus. The explanation must be sought in the disposition on the part of many to link all serious functional disorders with positive organic lesions, and on an imperfect acquaintance with the broad facts of pathology. Mr. Curling goes into an elaborate argument to shake the inflammatory hypothesis we have alluded to, but we do not consider it necessary to pursue him.

We must not forget to observe that actual inflammation of the spinal cord or of its membranes may occasion tetanus. This is not surprising. We have seen lesions of the brain give rise to it—we shall see lesions of the nerves occasion it—in the majority of instances mere functional disturbance or some very subtle change indeed of the nervous system produces it—and we cannot wonder if inflammation of the cord should do so. But such cases must be separated from the ordinary instances of tetanus, from which they differ in symptoms, as pathology. Take an example of tetanus from inflammation of the cord.

*Case.* A boy was struck on the back part of the neck by a shutter falling upon him, and was picked up insensible. On the following day symptoms of tetanus showed themselves, accompanied with a hot and dry state of the skin, the bladder became paralysed, the fæces passed involuntarily, and he died on the fourth day after the accident.—On examining the body, it was found that the cervical portion of the spinal cord, to the extent of an inch, had undergone considerable softening, and that the membranes covering this portion were inflamed and much thickened.

Sir B. Brodie has seen three cases of opisthotonos following injuries of the head, in all of which matter was found on the medulla oblongata. Other instances of inflammatory tetanus have been related, and are quoted by our author, but it is unnecessary to refer to them.

" In some few instances blood has been found extravasated within the spinal sheath. This was noticed in a case of idiopathic Tetanus recorded by Dr Funk. The effusion extended from the first dorsal vertebra to the lower part of the canal, being greatest in the lumbar region. It is stated, that the surface of the cord was rose red—the origins of the nerves swelled—and the cauda equina much reddened. In another case, of traumatic Tetanus in a female, mentioned by the same author, a similar appearance was observed." 63.

Other morbid changes of an anomalous character have been reported. In a child, aged between 3 and 4 years, who died in the Hôpital des Enfans Trouvés at Paris, with symptoms of opisthotonos, trismus, difficult deglutition, and coma, Ollivier discovered deposition of a red and very consistent fluid in the cellular texture between the dura mater of the cord and the bony canal of the spine in the dorsal region, serous effusion within the membranes, and the arachnoid of the medulla covered with an albuminous concretion for four inches. In a man, a large quantity of viscid yellow serum was found under the outer covering of the spinal cord. In the museum of St. Thomas's Hospital there are two preparations of bony and cartilaginous deposits beneath the arachnoid covering the medulla spinalis, in the dorsal and lumbar regions, distinct, small in size, and of the thinness of paper, which are described as having been taken from the bodies of patients who had died of tetanus. In case 94, a number of small bony patches were observed on the pia mater of the cord. In case 96, three small laminæ of bone were found on the dorsal division of the cord. Mr. Travers has often found such deposits. Our author has never found them. It is clear enough that they are not essential, though possibly, they may predispose to the occurrence of tetanus.

Such, then, is the morbid anatomy of the brain and spinal cord, so far as this disease is concerned. Nothing can be more vague. What we find is just enough to convince us that it is not necessary to find any thing. In the mass of cases, the only alteration is probably one of the vascular system of the brain and cord, an alteration which leaves no material traces after death, and is little under our control during the patient's life.

*The Sympathetic System.*

Mr. Swan, Dr. Aroussohn, of Strasbourg, Andral, and M. Dupuy, have directed attention to the state of the cervical ganglia of the sympathetic. They have found, or thought them inflamed. These appearances are even more suspicious than those of the brain and of the cord.

*The Nerves immediately connected with the Seat of Injury.*

There can be no doubt that, in numerous instances, the nerves of the injured part have been found inflamed or altered in their organization. This is a point of some little practical consequence, because whatever tends to localize the cause of the disorder is, of course, important. We quote the following summary of stated facts.

" In case 5, on examining the wound at the back part of the thigh, which was inflicted by a spike that had penetrated deeply into the semitendinosus muscle, driving a piece of wadding before it, I found the sciatic nerve, which passed close to the bottom of the wound, highly injected with blood. In case 4, I traced the internal plantar nerve to the wound, which was occasioned by a compound dislocation of the great toe, where it appeared thickened, and its neurilema unusually vascular. In the dissection of a patient of Mr. Ewbank, who had died of Tetanus after a wound of the leg by a pitchfork, the prong was found to have penetrated to the peroneal nerve, which was bruised, and implicated in the inflammation set up in the part. In case 64, after a laceration of the hand, which was amputated by Mr. Liston as soon as the tetanic symptoms made their appearance, the branch of the median nerve, going to supply the thumb, was found torn two-thirds across, and its extremity inflamed and thickened for nearly an inch. On dissecting the stump of a young officer, who died of Tetanus after the amputation of his arm, Baron Larrey and M. Ribes found the median nerve included in the ligature upon the artery, and its extremity swollen and red. In another case, mentioned by Larrey, the disease was consequent upon a wound by a bullet, which had passed through the internal and posterior part of the arm of a French general, cutting across the radial and internal cutaneous nerves and a portion of the biceps. Baron Dupuytren discovered, in the arm of a boy who died of Tetanus, a few days after being struck by a coachman, a portion of a whip enveloped in the very substance of the cubital nerve. In case 89, the internal plantar nerve, before it divided into the two branches which supply the great toe and the toe next to it, was completely torn through, and each extremity of the nerve was bulbous and vascular: every other part of the nerve appeared perfectly healthy. In case 13, the plantar branch of the tibial nerve was involved in the wound. Dr. Hennen mentions that in one case he found the radial nerve, connected with the injury, thickened, and a spiculum of bone sticking in it. Mr. Morgan found, on dissecting the thumb, in a case of Tetanus, two pieces of splintered teak, imbedded in the abductor muscle, and resting upon a branch of the radial nerve, without any appearance of inflammation in the part. In another case the sheath of the nerves supplying the injured part was highly inflamed." 68.

It is to be observed that injection of a nerve or of its cellular sheath, where neighbouring tissues are inflamed also, as after compound fractures, can hardly be considered satisfactory. Similar injection has been found, and no doubt would be often found, in cases where tetanus has not occurred at all. But where the nerve is actually thickened, or positively implicated in some way or other, the evidence is more satisfactory, and the fact deserves attention.

M. le Pelletier, chief surgeon at the Hospital at Mans, has communicated to the Royal Academy at Paris, a memoir in which he has attempted to show, that in all cases tetanus originates in inflammation extending from the neurilema of the nerves of the part injured to the membranes or substance of the medulla spinalis. In a case where tetanus occurred, after a compound fracture of the humerus, he found the neurilema of the cubital and median nerves red and inflamed, as also the envelopes of the brain and spinal marrow; and it was remarkable, that the appearances of inflammatory action were confined to the left side of these membranes, being the side corresponding with the arm that was fractured. This gentleman relates two other cases of a similar description, and further instances are referred to by Mr. Curling. But he very properly objects to receiving increase of vascularity, itself very dubious, as conclusive evidence of inflammatory action. In fact, in the case of the nerves, as of the nervous centres, we are under the necessity of owning, that the positive facts are few and far between, and that their rarity tends to throw a doubt upon their own importance.

### II. *Lesions of the Muscular System.*

The violence of the muscular contractions would be thought likely, à priori, to give rise occasionally to their rupture. Such is actually the case.

"We find that in case 28 the fibres of the psoas magnus were discovered after death lacerated and soft, blood being effused on the surface of this muscle and in the sheath of the recti-abdominis, the fibres of which were also torn and quite soft. In case 4, an account of which will be found at a subsequent page of this essay, I found, after death, that about half the fibres of the lower division of the rectus abdominis on the left side had been ruptured, the upper portion of the lacerated muscle being retracted, swollen, and hard. Several of the fibres of the right rectus were also torn in the same situation. A little extravasated blood was found in the sheath of these muscles at the seat of the rupture, which most probably took place in an acute and painful paroxysm that occurred just previous to dissolution. Baron Larrey relates the particulars of a very acute case of traumatic Tetanus, in which, during life, at the moment that the patient was plunged into a cold bath, a tumour, of the size of a hen's egg, was suddenly produced on one side of the linea alba below the umbilicus. It terminated fatally; and, on dissection, the right rectus muscle was found completely ruptured; and the swelling, which did not disappear after death, was formed by the upper portion of the muscle being retracted and turned in upon itself. Below this a rent was perceived filled with dark-coloured coagulated blood. The rupture had evidently taken place in the severe paroxysm induced by sudden immersion in cold water." 76.

The spasmodic contractions of the muscles have even caused fracture and dislocation of the bones. Desportes gives a case where both thighs were broken by the powerful action of the flexor muscles during a momentary remission of the extensors; and in a case mentioned by Fournier-Pescay, the spasms are said to have occasioned a dislocation of the second cervical vertebra.

III. *Lesions of the Viscera and other Parts of the Body.*

The lungs are often found gorged with blood, and sometimes in a state of *engouement*. The asphyxia which so frequently cuts off the patient accounts for these phenomena.

In all the cases in which Mr. Swan found unusual appearances in the sympathetic system, there was increased vascularity, and an inflammatory state of the mucous membrane of the alimentary canal. Dr. M'Arthur has published the details of four fatal cases of tetanus, in the dissection of which he discovered inflammation of the stomach and of the intestines. Andral relates a case, in which, on dissection, he found unequivocal marks of gastritis. In many cases worms have been found in the intestinal canal—ascarides sometimes, but more frequently lumbrici. In all probability the occurrence was accidental, yet they may be sought in investigations after death.

Enlargement of the papillæ maximæ at the root of the tongue and injection of the mucous membrane of the larynx, the pharynx, and œsophagus, are not unfrequent appearances in tetanus as in hydrophobia. The spasms and irritation of the parts explain, or seem to explain them.

The consideration of the theory of tetanus occupies forty-four pages. They shew what that theory is not—what it is they leave as unexplained a mystery as ever. We need not dodge the devious spirit of speculation, nor mention hypotheses for the mere purpose of exhibiting their hollowness. There are only two points, themselves scarcely theoretical, to which we consider it necessary to allude. They are the following.

1. It is a popular belief that wounds of the hands and feet are more likely to occasion tetanus, than when inflicted on other parts of the body. The reason has been supposed to be, that these parts are liberally supplied with nerves possessed of extreme sensibility. Now in the cases in the table it appears, that the original wound was on some part of the extremities in 110 instances, and in 69 either on the hands or on the feet. But it must be remembered that these parts are particularly exposed to injury. A numerical statement is presented of the location of a certain number of accidents treated at the London Hospital between the 23d of June, 1836, and the 26th of August in the same year. Of 510 cases, 193 were injuries on the upper extremities—124 injuries on the lower—86 wounds on the head—107 injuries to other parts of the body. Whatever influence may be exerted by the nerves of the part, it is certain that large nerves may be injured without the supervention of tetanus, and the liability to this is by no means in the ratio of the severity of the wound. Very severe ones may not occasion it, and the most trivial ones may.

2. It is an object of some moment to determine, if it be possible, when the disease becomes independent of its local exciting cause. Unfortunately, in the case of tetanus, as of hydrophobia, the period at which the irritation *has been* transmitted to the nervous centre, or the poison actually absorbed is little more than vague conjecture. On the one hand, we know that when tetanic symptoms have commenced, amputation, excision of the wounds, &c. have been often ineffectually performed—and, on the other hand, we have such a case as the one detailed by Mr. Travers. That gentleman states, that a man who had suffered amputation of the leg, above the knee, was attacked with violent tetanic spasms. These increased, until it was deemed expedient to open the stump and examine the ligatures, when it was discovered that the anterior crural nerve had been included in one of them: it was removed, when the spasms gradually subsided, and the man did well. Larrey mentions some similar cases, but the latter, perhaps unjustly, is scarcely deemed a very safe authority.

We now jump to a summary of what has either been established, or is probable in the way of theory. Yet we hardly know if the term theory will apply to some of the following deductions which constitute the summary in question.

"I am induced," says Mr. Curling, "to infer:—

1. That Tetanus is a functional disease of the nervous system, that is to say, a disease unaccompanied with any perceptible lesion of structure, the nature of which, although essentially distinct from inflammation, is completely unknown. There are, therefore, no morbid changes peculiar to Tetanus, and by which it can be recognised.

2. That the seat of this peculiar morbid action, termed *tetanic irritation*, is the *tractus motorius*, on either side, wholly or in part, the superior portion being the most generally affected.

3. That the result of tetanic irritation in the tractus motorius, or medulla, is a supply to the muscles of a stimulus to abnormal action, which, although limited to the muscles subservient to the will, is, nevertheless, totally without its control.

4. That tetanic irritation is excited in two ways; *first*, by a noxious impression propagated to the medulla from distant nerves, (most probably sentient) which impression may be caused by a wound, cold, or any other source of irritation; *secondly*, by inflammation in the brain, spinal cord, or their investing membranes, either idiopathic or occasioned by direct injury to these structures, or extending continuously from the nerves of a wounded part of the medulla. Traumatic Tetanus, commonly arises in the first way, its origin in direct injury to the cord, or in inflammation extending from an injured nerve, being exceedingly rare.

5. That, in pure traumatic Tetanus, the primary impression is confined for an indefinite time to the nerves of the part injured, being transmitted at some subsequent period to the medulla, and thus exciting the disease.

6. That when tetanic irritation is once fully excited in the medulla, which is made manifest by spasmodic contractions in the muscles, the disease is independent of the exciting cause, and does not cease upon its removal.

7. That the nervous system in some individuals is more disposed to take on this morbid action than in others, and that, as a general rule, males are more susceptible of it than females and negroes than whites.

8. That certain morbid states, as disorders of the digestive organs, the influence of particular climates, and a deleterious atmosphere, render the system more susceptible of this disease.

9. That the derangement of the vital organs in Tetanus is the result of the inordinate action of the voluntary muscles induced by the disease, the disturbance and suspension of different functions, and even fatal exhaustion, being chiefly, if not solely, referrible to the violent muscular contractions. In fact, the tetanic irritation directly interferes with, or affects, no organ nor part whatever besides the system of the voluntary muscles.

10. That tetanic irritation often gives rise to a determination of blood to the cord and its meninges, and to the nerves proceeding from the site of the wound and to the affected muscles, the result of which, in the medulla, is an increase in the natural secretion of the arachnoid. The minute vascular injection of the cord and of the nerves, together with the serous effusion at the base of the brain and between the spinal membranes, being therefore nothing more than occasional effects of the disease, are by no means constantly present after death." 123.

### TREATMENT OF TETANUS.

A statistical view of the treatment of tetanus is not so useless as, at first sight, it may appear. Such a view shews what has been done, and what has not been done—what has proved of some advantage, and what has proved of none. But it cannot be received as offering the means of establishing any ratio of benefit from remedies, for there can be little doubt, that the unsuccessful cases have not been duly published. If a remedy has succeeded or appeared to succeed, the case has, no doubt, been placed on record. If the same remedy has failed, the details have, too often, never seen the light.

### 1. *Cure without any Treatment.*

This has occurred. It is wonderful, not because there was a cure, but because there was no treatment. Probably a fair proportion of the cures *with* treatment have been in spite of it. But to return. Dr. Morrison notices a well-authenticated instance of a negro, who recovered from the disease without having recourse to any remedies. Mr. Morgan mentions a case that occurred at Guy's Hospital, in which, although the patient was left entirely to nature, the disease ran its course, and eventually the man recovered; and Sir Astley Cooper states, in his lectures, that he has seen similar cases.

### 2. *Treatment of the Premonitory Symptoms.*

It is important to recognize the premonitory symptoms of tetanus, because it is possible we may exercise a control over them, which we have not over the disease when it is established. What are those premonitory symptoms?

Twitchings of the muscles of the affected limb, a degree of lassitude, restlessness, great depression of spirits, and an uneasy sensation about the præcordia, are often observed previous to its development. But the most common precursors of both forms of tetanus are cold chills, and an uneasiness about the throat, leading the patient to imagine that he has caught cold. These latter symptoms are frequent enough to render it important for the surgeon to bear them in mind when attending the subjects of injuries, especially the wounded after a battle.

It is better to be on the safe side and to regard such symptoms with suspicion.

### 3. *Local Treatment.*

The disease being the consequence of local irritation it was natural to think that the removal of it would remove, or tend to remove its effect. The means adopted may be looked upon as essentially two: 1. Amputation or excision of the wounded part; 2. Division of the nerves proceeding to the seat of injury.

#### *Amputation.*

It is obvious that the earlier this is performed the better must be the chance from it. It was resorted to in eleven of the cases in the table, seven of which were cured. In case 14, it was employed in conjunction with tobacco soon after the symptoms were fully established. In case 23, related by Mr. White, of Manchester, the symptoms were somewhat advanced, severe but chronic, and opium was freely employed. In case 55, the original wound was of itself sufficiently severe to demand amputation; the operation was performed on the day after the appearance of symptoms, and it is stated that "the tetanic symptoms were evidently kept under and finally removed, chiefly by the use of opium." In case 61, the symptoms were exceedingly severe. The particulars of it are recorded by Mr. Wayte, a surgeon at Calne. The patient was a boy, eight years of age, and on the day after the appearance of tetanus, which was consequent upon a severe burn on the arm from lime, the limb was amputated. The symptoms subsided gradually but uninterruptedly, and, in little more than a month, he was perfectly recovered. No other remedies were employed, except one anodyne draught before the appearance of tetanus, and occasionally a mild purge. In case 77, treated by Mr. Howship, the primary wound was a severe injury to the elbow-joint, the symptoms having been mild, and of slow access, and not well marked, and amputation was not resorted to till the third day after they appeared. Case 99 is the instance alluded to by Larrey. In case 119, the patient was a female, and the operation was not resorted to till the thirteenth day after the attack, when the symptoms were subsiding under the influence of opium. Dr. Huck, physician to the army in the year 1758, in a case of tetanus occurring after a fracture of the metacarpal bone that sustains the forefinger, by a musket ball, on the day following the appearance of trismus, amputated the finger, and removed the sharp points of the remainder of the metacarpal bone. Opium was afterwards given, and the patient recovered.

These cases, tend rather to favour the practice of amputation of the injured part. Yet there are many facts of an opposite tendency to be arrayed against them. Sir James M'Gregor mentions, that after the battle of Toulouse amputation was extensively resorted to without affording relief; and Dr. Hennen and Mr. Guthrie were equally unsuccessful with the same method of treatment. Dupuytren strongly objects to the performance of amputation; and Sir Astley Cooper alludes to the trial of it in three instances, which were all fatal. Mr. Grimstone mentions two cases where amputation was performed on the first appearance of symptoms, but they terminated fatally. Sir Benjamin Brodie unintentionally made the experiment of amputation on a boy who was admitted into St. George's Hospital with compound fracture of the leg, which was followed by gangrene of the limb. The operation was performed while the gangrene was spreading; but subsequently it was ascertained, that the patient had complained of the premonitory symptoms of tetanus the morning before the operation. The disease was rapidly developed after the limb was removed, and the boy died in less than twenty-four hours. We witnessed this latter case, and another in St. George's Hospital, where amputation of the thumb was performed without advantage, after the commencement of the tetanus. On the whole, it must be owned that the balance of evidence is against the operation.

*Division of the Nerves.*

This was first practised with success by Mr. G. Hicks, surgeon of Baldock, in 1797. The case was one of trismus, not acute, which followed a contusion in the palm of the hand. The issue was successful. Two other successful cases are detailed by Baron Larrey, and another by Dr. Murray. These four are the only unexceptionable cases in which, so far as our author knows, the operation was performed. They do not prove much, but the practice merits in appropriate cases further trials.

4. *Constitutional Treatment.*

A. *Purgatives.*—These have been much used, partly because there is usually obstinate constipation—partly because depraved secretions have been brought away by them. The purgatives have been of all kinds. In case 48, a single drop of croton oil procured copious evacuations. In case 27 two drachms of calomel, sixteen drachms of jalap, and fifteen grains of scammony, were taken, but the bowels were not relieved for three days; and afterwards, forty grains of calomel, fifty-one grains of the extract of colocynth, and thirty-eight of gamboge, were taken daily during nine days, in order to keep up their action.

After purgatives have been employed, depletion and all kinds of sedatives and antispasmodics have been liberally had recourse to. We all know what they have failed to do. Mr. Curling treats separately of some of the remedies that have enjoyed most reputation.

B. *Mercury.*—This was first recommended by Mr. Donald Monro, on the authority of a physician who had used it with great success for idiopathic tetanus in the West Indies. Yet the evidence of Sir James M'Gregor, Baron Larrey, Dr. Wells, &c. is against it. Mr. Curling saw it tried without the least benefit in two cases, in both of which much suffering was occasioned by the salivation. Of fifty-three cases in the table, where mercury was employed, thirty-one proved fatal. Of the twenty-two cases which recovered, in case 57 it is mentioned, that although salivation was produced, the symptoms continued to increase until the quantity of opium was augmented. In case 80 mercury was not resorted to until the disease had been established seventeen days; and in another, case 90, the patient was improving before ptyalism was produced. In twenty of these cases of recovery opium was combined with this remedy; and of the two treated without it, in one tobacco injections were employed. Six of the cases also were of females. In eleven of the cases, in which neither opium nor tobacco was resorted to, the mercury being given alone, or employed in conjunction with some trivial remedy, as the warm bath or blisters, *all were fatal* except one. Twelve cases of tetanus, consequent upon severe injuries, are related by Mr. Howship in the London Medical and Physical Journal, in all of which mercury was freely exhibited. Two only recovered, and in both of them it was given in conjunction with opium. This evidence is pretty unequivocal against it. When tetanus depends on inflammation of the cord or of its membranes, mercury combined with opium, becomes a very proper remedy.

C. *Blood-letting.*—This is proper enough where the symptoms are decidedly inflammatory. Where they are not, facts are on the whole against its employment. Mr. Guthrie tried venesection in three cases of traumatic tetanus. In one, in which the disease had advanced with rapidity, the patient was bled nearly *ad deliquium* several times with good effect, calomel and diaphoretics being given at the same time: he recovered. In the second case, the patient was bled in the same manner, with an evident amendment of the symptoms, when he was seized with diarrhœa, which, in his debilitated state, carried him off. In a third case of a man of sanguine temperament, and suffering from acute tetanus, venesection, pushed to the utmost, totally failed. Of twenty-six of the cases, in which blood was abstracted and other remedies also resorted to, thirteen died. Two other cases in the table, unattended with inflammatory symptoms, were treated by bleedings alone; both terminated fatally. In case 28, the pulse was full and strong, beating about 140 in a minute, Mr. Earle bled the patient repeatedly, and, after every venesection, there was decided remission of the spasms. The blood was remarkably buffed and cupped; he seemed to be in a fair way for recovery, when the disease was, upon two occasions, aggravated by the friends giving him, most injudiciously, wine and porter. He died afterwards, apparently from exhaustion.

Loss of blood appears to favour the operation of medicines in this disease, as in other affections. Thus, in case 87, the patient being of a full plethoric habit, bleeding seemed to assist the action of opium. If blood-letting is to be resorted to at all, it should be in appropriate cases, and at an appropriate time. But it is a dangerous remedy.

D. *Counter-irritation.*—Unless the case is inflammatory, this is of no service, and plagues the patient.

E. *Opium.*—It is unnecessary so say how much this has been used. Opium, in various forms, and in conjunction with other remedies, was employed, in eighty-four of the cases in the table, being more than two-thirds of the whole number. Of these, forty-five recovered, of which ten were females. Yet there can be no doubt that the confidence of surgeons in its powers is not great. The quantities that have been given in particular cases are not trifling.

"In Tetanus, as well as in hydrophobia and delirium tremens, there appears to be a conventional license for the unlimited exhibition of opium. A drachm, and two drachms of laudanum, or a scruple of the solid opium every three or four hours, are ordinary doses. In a case related by Mr. Leath, in the London Medical and Physical Journal, ninety-nine ounces, seven drachms of laudanum, were taken in little more than a month, the quantity exhibited in the course of the twenty-four hours for eleven days having been three ounces, six drachms. Begin, in his Traité de Therapeutique, relates that M. Blaise, in a case of Tetanus, administered in ten days four pounds, seven ounces, and six drachms of laudanum, and six ounces, four drachms, and forty-five grains of solid opium! It cannot but excite surprise that such enormous doses of this drug, both in substance and in solution, should have been taken internally in these cases, without in the slightest degree controlling the symptoms of the disease, or producing upon the system any of its ordinary effects. There is, however, reason to believe that these large doses have not always been received into the system. Mr. Abernethy, on opening the stomach of a patient who had died of Tetanus, after taking largely of opium, found thirty drachms of this substance undissolved in the stomach. In two fatal cases of delirium tremens I found in the stomach of each patient after death several drachms of laudanum and opiate pills, obviously unchanged or unacted upon." 152.

Where opium cannot be swallowed, it may be exhibited in the shape of glyster. Dr. Latham has recommended Dover's powder. Dr. Stutz, of Suabia, has cured three cases by opium combined with alkalies, and warm baths impregnated with the deutoxyde of potassium and lime.

F. *Tobacco.*—There appears to be stronger evidence in favour of this, than of any other remedy. Mr. O'Beirne has particularly insisted on its efficacy.

"Of nineteen cases in the table, in which tobacco was employed, nine recovered. Of the ten fatal cases it must be remarked, that in case 20 the remedy was not resorted to till the patient was dying. In case 28, treated by Mr. Earle, and in case 49, the case of Dr. Anderson's just alluded to, it would appear that this remedy was not fairly tried. In case 39, there was organic disease in the brain, and in case 52 the symptoms were always moderated after the injections; but the patient was not kept constantly under its influence. Case 53 was remarkably severe, the disease having proved fatal in forty hours, and the remedy was scarcely resorted to in time. In neither of the two cases (cases 105 and 106) treated by Mr. Carmichael, does it appear that the tobacco was exhibited to an extent sufficient to produce that powerful impression upon the system by which alone the spasms in the acuter forms of the disease can be combated." 168.

Two other cases were watched by our author at the London Hospital. In the first the patient died from the depressing influence of the narcotic. In the second he died asphyxiated. The latter was on several accounts not a fair case. In the former sufficient caution was not used. On the whole, Mr. Curling remarks that he has not succeeded in finding a single case, in which, being fully and fairly tried before the powers of the constitution had given way, it has been known to fail. Of course great care must be used in exhibiting this powerful drug. Its dose must be proportioned to the age, strength, and state of the patient, and its depressing influence should be counteracted by appropriate stimulants.

G. *Antimony.*—Of ten cases in the table in which it was resorted to, six were fatal, but in these the disease was very acute. In a case of chronic tetanus treated by Mr. Liston, it seemed serviceable, as it did in a case of idiopathic tetanus detailed by Mr. Woodward.

H. *Cold Affusion.*—This was first recommended by Hippocrates—has been much practised in the West Indies—and has met with a certain degree of favour from many surgeons. Its effects are by no means slight. The immediate consequence is often a severe and sudden exacerbation of the spasms, as in case 46, which sometimes proves fatal, as in an instance recorded by Baron Larrey. Mr. Morgan likewise mentions a case, where the patient was plunged, by his own desire, into a cold bath; all the symptoms of tetanus disappeared in a moment, and he was almost instantly taken out lifeless. The shock produced on the system, by the impression of cold upon the surface of the body, had occasioned instant death. In a severe case of tetanus, described by Dr. Currie, the patient was plunged into cold water, at 36° Fahr. When taken out the pulse and respiration were almost entirely suspended. Warm blankets, however, had been prepared, and general friction was diligently employed. The respiration and pulse became regular, the vital heat returned, the muscles continued free from spasm, and the patient fell into a deep sleep. In this case a single impression was sufficient to arrest the disease. In case 120, its immediate effect was to suppress respiration and to extinguish the pulse at the wrist and the heat of the surface of the body. In case 90, during its operation, the pulse was reduced from 150 to 90.

The evidence in favour of the cold affusion refers chiefly to idiopathic tetanus. Mr. Samuel Cooper says that it presents no hope of benefit in cases of traumatic tetanus. But Mr. Samuel Cooper is clearly wrong, for not only *may* hope be indulged from its use, but it has actually effected, or seemed to effect a cure. Thus Sir Benjamin Brodie has seen it of more use than any other remedy. Of twelve cases in the table, in which cold affusion and other means were adopted, seven terminated favourably. Independently of those cases, three others have been collected by our author, in which cold affusion was of signal service, the patient in each instance recovering. A case communicated to Sir James M'Gregor by Mr. Nixon, Deputy Inspector of Hospitals, appears to illustrate the sedative and beneficial influence of cold. It occurred in the march of the guards through Gallicia.—The symptoms of tetanus arose from a slight wound of the finger, and are stated to have been unusually severe. As it was impossible to think of leaving the man in the wretched village where he was attacked by the disease, he was carried on a bullock-car after the battalion. During the first part of the day he was drenched with rain, the thermometer standing at 52°, but, after ascending one of the highest mountains in Gallicia, the snow was knee deep, and the thermometer below 30°. The patient was exposed to this inclement weather from six o'clock in the morning till ten at night, when he arrived, half starved to death, but perfectly free from every symptom of tetanus. This man was, therefore, solely indebted for his recovery to the sedative influence of intense cold, to which he was accidentally subjected.

I. *The Warm Bath.*—This appears to be useless, if not dangerous in acute cases, may be serviceable in chronic, and has been beneficial in removing the muscular stiffness left after tetanus.

K. *The Vapour Bath.*—This would seem to have been successful in two out of three cases reported by Dr. Marsh of Dublin, and in two cases related in the Journal Hebdomadaire.

L. *Tonics and Stimulants.*—In the exhaustion occasioned by tobacco, or indeed by the disease, these are serviceable, nay, necessary. The extent to which they may be carried, if not required, is shewn in a case related by Dr. Currie of Liverpool. A gentleman was seized with tetanus, which was excited by a splinter having been driven under the nail of his middle finger. In less than a month, he drank, mixed with nourishment, 140

bottles of Madeira wine, finishing four or five in the twenty-four hours, besides ale and brandy, two gallons of strong broth, and about two drachms and a half of laudanum. Laudanum and æther were also freely used as embrocations; and, in addition to this, he bathed altogether sixty-five times, the water being from 60° to 64° of Fahr., by which he was always relieved. His previous habits, in regard to the use of wine, are not mentioned; but it is stated, that he was in the vigour of life, of great bodily strength, and that, during the paroxysms, the sweat poured in torrents from his body.

M. *Carbonate of Iron.*—This has been much recommended by Dr. Elliotson.

" In two severe cases (33 and 34), after the bowels had been freely acted upon by turpentine, he gave the carbonate of iron, in doses of half an ounce, every two or three hours: both terminated favourably. In a third case, the disease was far advanced when the remedy was resorted to, and the patient died, within thirty hours afterwards, in a violent paroxysm. Another case, (17) treated with the carbonate of iron, at the Brighton Hospital, also ended fatally. In a fifth case, (21) in which it was tried by Dr. Dehane, of Wolverhampton, the symptoms were severe, and, in the first instance, bark, ammonia, and laudanum were resorted to; but, on the fourth day, no amendment having been produced, the carbonate of iron was given, to the extent of 1lb. daily, under which treatment the spasms gradually subsided. It was remarked, in this last case, that, on the fifth day after the iron had been used, the patient having voluntarily discontinued it, the spasms were increased, but they slowly subsided as soon as it was resumed, I am not acquainted with any other case in which this remedy has been tried." 197.

N. *Hydrocyanic Acid.*—This has been tried in a few cases, but with results not at all calculated to encourage us.

O. *Other Remedies.*—The colchicum autumnale, injection of opium and stramonium into the veins, inoculation with the ticunas, have been tried with a little dubious advantage.

In conclusion, Mr. Curling again insists on the necessity of discriminating between three forms of tetanus, which exhibit material practical differences—the pure acute tetanus—acute inflammatory tetanus—and chronic tetanus.

His principles of treatment in the first consist, in maintaining a free action of the bowels, in allaying the spasms with the tobacco, cold affusion or any other sedative equally effective, and in the due exhibition of tonics and stimulants.

For the second, antiphlogistic treament is necessary, combined with the means for allaying the spasms indicated in the first case.

For chronic tetanus he thinks the following remedies may be variously resorted to, according to the particular circumstances of the case. Purgatives, opium, antimony, vapour and warm water baths, the carbonate of iron, and other tonics, electricity. If the case be attended with febrile or inflammatory symptoms, proper means must be taken to remove them. In such instances the exhibition of opium, and other sedatives, may often be advantageously combined with antiphlogistic treatment; and it should be borne in mind, that although the spasms may be partly allayed by depletion, their complete removal does not depend upon the extent to which it is carried; and that the debility resulting from active depletion, is invariably prejudicial to ultimate recovery from tetanic irritation.

Appended to the volume is the table to which reference has been so often made. It is far too valuable to be omitted here, and will serve as a frequent source of reference to those who are anxious to become acquainted with the most authentic facts we possess respecting the disease. Long as is the table, we therefore extract it.

[*See Table on the following page.*]

A formal eulogy of this unpretending but very excellent book would be superfluous. The use that we have made of it, the space the present article occupies, assure our readers that we entertain a very high opinion of the industry and judgment of its author. Itself, for the most part an analysis, we could not analyse it. We have been compelled to extract rather than abridge, for who could much abridge summaries of facts?

The book should be in the library of every surgeon and physician. It is a valuable work of reference. It does not pretend to originality, for originality on such a subject was not wanted. But a compendium of facts *was* wanted, and such a compendium is this volume. We cannot part from Mr. Curling without thanking him for the information we have received in reading his work, and for the matter it has enabled us to offer to our readers.

TABLE OF ONE HUNDRED AND TWENTY-EIGHT

*Showing the Sex, Age, nature of the Injury, at what period after the Wound Tetanic Symptoms, the Remedies resorted to, and the*

MA

| No. | Name. | Age. | Nature of the Injury. | Appearance of Symptoms. |
|---|---|---|---|---|
| I | —— | 10 | Gun-shot wound of the calf of the right leg | 5th day |
| II | T. Mountford | 21 | Forefinger of the right hand injured by a nail | 3 weeks |
| III | —— | 30 | Severe contusion of two of the fingers of the right hand | A fortnight |
| IV | Thomas Moss | 22 | Compound dislocation of the great toe of the right leg | 10 days |
| V | Sand. Philipps | 42 | Right thigh wounded by an iron spike | 9th day |
| VI | —— | 26 | A blow on the head, and thrown into a canal | 4th day |
| VII | —— | 19 | Sole of the left foot injured by a nail | 3 weeks |
| VIII | —— | 31 | Compound dislocation of the astragalus and os naviculare | 7th day |
| 9 | Thos. Purkiss | 40 | Contused knee | 9th day |
| 10 | William Kidd | 16 | Laceration and contusion of the hand | 13th day |
| 11 | Wm. Golden | 16 | A blow on the back of the neck | Follow'g day |
| 12 | J. Thompson | 25 | Lacerated wound of the little toe | 10th day |
| 13 | W. Hayes | 14 | Sole of the foot wounded with an iron spike | 11 days |
| 14 | H. S. | | Forefinger wounded with a blunt piece of tin | 12 hours |
| 15 | L.—— | 21 | Wound in the sole of the foot with a nail, followed by an abscess | 16th day |
| 16 | —— Cavazo | 33 | Foot slightly wounded with a nail | A month |
| 17 | Jas. Coppard | 70 | Contusion of right hand, with fracture of three of the metacarpal bones | 10 days |
| 18 | Robert Gutter | 21 | Lacerated wound of the integuments of the leg closed with sutures | 16th day |
| 19 | William Pile | 15 | Foot wounded with a rusty nail | 11 hours |
| 20 | —— | 10 | Fracture of both bones of the right leg, with extensive laceration of the integuments | 9th day |
| 21 | Robert Ireland | 43 | Fractures of the left leg, crista of the ileum and great toe of right foot, with lacerations | 10th day |
| 22 | James Greaves | mid. age | Foot wounded with a nail | 14th day |
| 23 | Samuel Wood | 20 | Contused wound of the finger | A month |
| 24 | A. M'Farlan | 23 | Amputation at the shoulder joint, on account of a gun-shot wound of the arm | 26th day |
| 25 | Frank, a Negro | 22 | Contusions about the head and body | 3rd day |
| 36 | ——, a Negro | 25 | Sole of the foot wounded with a rusty nail | |
| 27 | Sam. Joyce | 35 | Contused wound on the forehead | 24th day |
| 28 | Joseph Owen | - | Laceration of the great toe | 3 weeks |
| 29 | J. Collins | 30 | Amputation of two fingers | 11 days |
| 30 | W. Merrit | 30 | Amputation of the leg | 13th day |
| 31 | W. Harris | | Amputation at the shoulder joint | 12th day |
| 32 | —— | 19 | Slight wound of the left hand | 18th day |
| 33 | Chas. Frasier | mid. age | Compound dislocation of the great toe | 14th day |
| 34 | B. Macquire | 44 | Right thumb jammed between two pieces of wood | 8 days |
| 35 | J. Flood | 13 | Feet torn by machinery | 9th day |
| 36 | H. Southerby | 14 | A bite by a dog in the leg | A week |
| 37 | —— | 15 | Both heels lacerated, and left humerus fractured | 7th day |
| 38 | —— | 22 | Sole of the foot wounded with a nail | 5th day |
| 39 | John Ashford | 40 to 50 | A blow on the side of the head | 4 weeks |

CASES OF TRAUMATIC TETANUS.

Symptoms were first evinced, the Results, and at what time after the Appearance of
Sources from which the Cases have been obtained.

LES.

| Result, and in what time after. | TREATMENT. | AUTHORITY. |
|---|---|---|
| Fatal, 16 hours .. | Purgatives. Opium. Tartar emetic bath .... | The Author.. *vide page* 31 |
| Fatal, 4 days ..... | Purgatives. Opiate injections .............. | —— .......... 88 |
| Cured, 3 weeks .. | V.S. Opium. Calomel .................... | —— .......... 143 |
| Fatal, a fortnight | Opiate injections. Stimulants .............. | —— .......... 158 |
| Fatal, 3rd day .... | Opium. Tobacco ........................ | —— .......... 169 |
| Fatal, 9th day .... | Mercury. Opium. Tobacco .............. | —— .......... 170 |
| Cured .......... | Purgatives. Opiate injections. Stimulants .... | —— .......... 194 |
| Fatal, 2nd day.... | Hydrocyanic acid ........................ | —— .......... 199 |
| Fatal, 6th day .... | Purgatives. Opiate injections ............ | —— |
| Fatal, 15th day .. | Purgatives. Opium. Stimulants .......... | —— |
| Fatal, 2nd day.... | C. C. Nuchæ. Croton oil. Opium. Belladonna | Medical Gazette, i. 645. |
| Fatal, a fortnight . | Purgatives. Mercury. Opium. Antimony. Musk. Turpentine. Belladonna. ...... | —— i. 646. |
| Fatal, 2nd day . .. | Cold affusion. Sulph of Quinine. Hydrocy. acid | —— ii. 343. |
| Cured, a few hours | Amputation. V.S. Tobacco. Opium ...... | —— ii. 141. |
| Fatal, 10th day .. | V.S. Leeches. Opium. Musk. Mercurial friction.......................... | —— vi. 830. |
| Fatal, 18 days.... | V.S. Leeches. Opium. Vapour-baths | —— vii. 312. |
| Fatal, 2nd day.... | Blisters. Turpentine injections. Carbonate of iron......................... | —— vii. 383. |
| Fatal, 7th day .... | Opium ................................ | —— vii. 828. |
| Cured, 3rd day .. | Division of the nerve. Opium. Camphor. V.S. | —— xi. 623. |
| Fatal, 2nd day.... | Nerve divided. Croton oil. Opium. Antimony. Tobacco. Cautery ......... | —— xii. 15. |
| Cured, a month .. | Bark. Opium. Carbonate of iron .......... | —— xii. 799. |
| Cured, 6 weeks .. | V.S. Musk. Opium. Valerian............ | Medical Obs. & Inq. i. 51. |
| Cured, 6 weeks .. | V.S. Blister. Musk. Bark. Camphor. Valerian. Amputation.............. | —— ii. 382. |
| Cured, a month .. | Musk. Opium ........................ | —— iii. 330. |
| Cured, a week.... | Cold affusion .......................... | —— vi. 148. |
| Cured, 4 days .... | Cold affusion. Opium. Bark.............. | —— vi. 155. |
| Cured, 5 weeks ... | Mercury. Colocynth. Gamboge. Scammony. Opium. Stimulants ......... | Med.-chir Trans. ii. 284. |
| Fatal, 7th day.... | V.S. Mercury. Dover's powders. Tobacco. | —— vi. 93. |
| Fatal, 2nd day.... | Opiate injections.......................... | —— vii. 468. |
| Fatal, 8th day.... | V.S. Warm-bath. Mercury .............. | —— vii. 470. |
| Fatal, 2nd day.... | Warm-bath. Opium........................ | —— vii. 473. |
| Cured, 24 days ... | V.S. Warm-bath. Purg. Merc. Dover's powd. | —— xi. 384. |
| Cured, 10 days ... | Turpentine. Carb. of iron. Musk. Stimulants | —— xv. 163. |
| Cured, 25 days ... | Turpentine. Carbonate of iron ............ | —— |
| Cured, 6 weeks ... | Purgative. Turpen. injections. Leeches. Tobacco | Dublin Hospital Rep. iii. 357. |
| Cured, a month .. | Purgat. Mercury. Opium. Leeches. Tonics | Bright's Medical Rep. ii. 558. |
| Fatal, 40 hours ... | Musk. Purgatives. Opiate injections ....... | —— 572. |
| Fatal, 4th day .... | Opium. Blisters. Antimony .............. | —— 573. |
| Fatal, 4 weeks.... | Purgatives. Tobacco. Turpentine.......... | —— 579. |

| No. | NAME. | Age. | NATURE OF THE INJURY. | Appearance of Symptoms |
|---|---|---|---|---|
| 40 | John Patrick . | 12 | A burn on the right thigh, penis, scrotum, and abdomen, | 10th day .. |
| 41 | Rich. Burton | 37 | Foot wounded with a spike .......................... | 3 weeks ... |
| 42 | —— | 17 | Severe contusion of the left heel .................... | Some days |
| 43 | —— | 44 | A wound in the leg .................................. | 15th day .. |
| 44 | —— Payne ... | | Finger pierced by a splinter ......................... | 10 days ... |
| 45 | James Sydling | 40 | Amputation of the leg ................................ | 5th day .. |
| 46 | Wm. Godby.. | 5¼ | Lacerated wound of the instep, and great toe fractured .. | 8th day .. |
| 47 | Wm. Collins . | 30 | Laceration of the integuments over the gastrocnem. muscle | 20 days.... |
| 48 | —— | 50 | Slight graze on the nose .............................. | |
| 49 | Smart, a Negro | 18 | Slight contusion of one of the toes .................. | 10 days.... |
| 50 | Luke, a Negro | 12 | Punctured wound of the thumb ....................... | Several days |
| 51 | ——, a Negro . | 22 | Slight wound of the head ............................ | 7th day .. |
| 52 | W.Gray, a Neg. | 28 | Thumb wounded with a splinter ........ | 9 days ... |
| 53 | —— | | Great toe punctured with a nail ........ . ......... | 2 weeks.. |
| 54 | Chas. Wattell | 30 | Right hand wounded by a pistol-ball .................. | 7th day .. |
| 55 | M. M'Gowan | 22 | Wound of the elbow-joint from a musket-shot.......... | 16th day .. |
| 56 | ——, a Negro | 20 | Laceration and contusion of the left foot............... | 15th day .. |
| 57 | Jas. Macrae .. | 15 | A severe lacerated wound of the left leg ... .......... | 10th day .. |
| 58 | Geo. Pybus .. | 27 | A wound on the left leg............... ............... | A fortnight |
| 59 | Robt. Millar.. | 20 | Two musket-shot wounds in the left arm .............. | A month .. |
| 60 | John Brown.. | 25 | A bite on the thigh .................................. | 2nd day .. |
| 61 | Robert Tilling | 8 | A severe burn on the right hand ............ ......... | 10th day .. |
| 62 | ——, a Negro | | Sole of the foot wounded with a rusty nail ............ | 13th day .. |
| 63 | Jos. Wishart.. | 20 | Contusion of the abdomen.............................. | 17 days.. |
| 64 | Wm. Boyle .. | 14 | Laceration of the hand ............................... | 12 days ... |
| 65 | Evan Wright | 15 | Severe compound fracture of the thumb................ | A fortnight |
| 66 | John Key.... | | Left arm wounded by a musket-ball..................... | 7th day .. |
| 67 | —— Gray .... | 16 | Arm shot away by a cannon-shot. Amputation at shoulder-joint five days afterwards ............ | 3 weeks.. |
| 68 | Elijah Dunn.. | 14 | An extensive wound on the right leg, and a slight wound on the outer ankle of the left leg ........ | 10 days ... |
| 69 | —— | 16 | A wound on the hip from a splinter...................... | 5th day... |
| 70 | W. Richey .. | 30 | Right arm shot away—amputation at the shoulder-joint | 8th day .. |
| 71 | M. Wheelan.. | 18 | Wounded on the shoulder and in several places on the back by splinters ..... .................. | 8th day .. |
| 72 | —— Robinson | mid. age | Compound fracture of the hand by a grape shot........ | 9th day .. |
| 73 | W. Meath ... | 30 | Leg shot away—amputation ............................ | 9th day .. |
| 74 | J. Cairns .... | | Wounded in the groin by a musket-shot.................. | 8th day .. |
| 75 | T. Kelly ..... | | A compound fracture of the leg ......................... | 10th day .. |
| 76 | W. Connars.. | 29 | A severe wound in the back by a splinter.............. | 11th day .. |
| 77 | Wm. Taylor.. | 25 | Elbow-joint shattered by a ball.......................... | 22nd day .. |
| 78 | Jas. Warring | 20 | Severe contusion of the thumb of the right hand ...... | 15th day .. |
| 79 | —— Landers.. | 23 | Sole of the foot wounded with a rusty nail ............ | 8th day .. |
| 80 | J. Milwood .. | | A wound upon the great toe from a hand-saw ........ | 10th day .. |
| 81 | Thos. Platt .. | 46 | Compound fracture of the thigh ................ .... | 3 weeks.. |
| 82 | J. Adams .... | 44 | Severe laceration of the hand and fingers............... | 10th day .. |
| 83 | J. Murdoch .. | 13 | Punctured wound of the leg ........................... | 9th day .. |
| 84 | J.C. Wright.. | 49 | Punctured wound of the thumb ........................ | 8th day .. |
| 85 | A. Brunetti.. | 58 | An incised wound on the back of the hand ............ | 21st day .. |
| 86 | Lewis Fonzi.. | 50 | Slight wound on the finger ............................ | |
| 87 | Fran. Mailhiot | 50 | Wounded with a nail in the sole of the left foot...... .. | 10th day .. |
| 88 | John Johnson | 20 | An incised wound of the extremity of the finger........ | 16th day .. |
| 89 | T. T. Mitchell | 20 | Wounded with a rusty nail in the sole of the foot ...... | 5th day .. |
| 90 | J. Kelly ..... | 14 | Fracture of the radius, and laceration of the integuments of the right leg.......................... | 16th day .. |

| Result, and in what time after. | Treatment. | Authority. |
|---|---|---|
| Fatal, follows day | Opium. Musk .................. | Swan, on Tetanus, 77. |
| Fatal, 6th day.... | Turpentine. Opium. Mercury. Amputation | —— 82. |
| Fatal, 20 days.... | .................................. | Morg. de Causis, &c. liv. 49 |
| Cured, 3 weeks ... | Opium. Stimulants. Cold affusion ......... | Currie's Med. Reports, i.169 |
| Cured, 8 weeks ... | Opium. Stimulants. Cold affusion ........ | —— i. 172 |
| Fatal, following day | V.S. .................................. | Parry's Cases of Tetanus |
| Fatal, following day | Opium. Cold affusion. Antimony .. ...... | —— |
| Cured, 10 days ... | Opium. Mercury .......................... | —— |
| Cured, 10 days ... | V.S. Purgatives. Opium ............... .. | Lawr.'s Lec. Med-Gaz. v.549 |
| Fatal, 3rd day.... | Purgatives. Tobacco........................ | Edinb. Med.-chir. Tr. ii. 366 |
| Cured, a month .. | Purgatives. Tobacco........................ | —— ii. |
| Fatal, 8th day.... | Mercury. Opium ......................... | Jamaica Phys. Journal, 1834 |
| Fatal, 5th day.... | V.S. Tobacco ............................. | —— |
| Fatal, 40 hours .. | Tobacco .................................... | —— |
| Cured, a month .. | Opium. Cold-bath. Digitalis. Stimulants .. | Edin.Med.& Sur. Jour. vii. 14 |
| Cured, a month .. | V.S. Opium. Amputation. Warm-bath. Mercury | —— viii. 412. |
| Cured, 6 weeks .. | Opium. Wath-bath. Tobacco. Stimulants.. | —— xi. 198. |
| Cured, 3 weeks .. | Mercury. Opium. Stimulants. Tonics .... | —— xi. 306. |
| Cured, 6 weeks .. | Opium. Stimulants. Tonics ........... .. | —— xi. 422. |
| Cured, a month .. | Opium. Stimulants ......................... | —— |
| Cured, a fortnight | Purgatives. Opium. Mercury. Stimulants.. | —— xiii. 451. |
| Cured, a month .. | Amputation................................. | —— xvii. 394. |
| Fatal, 20 hours .. | Mercury. Opium. Camphor ........... .. | —— xviii. 38. |
| Cured, a month .. | V.S. Opium. Blisters. Mercury.......... | —— xviii. 191. |
| Fatal, 10 days.... | Amputation. Opium. Mercury............. | —— xxi. 292. |
| Cured, a fortnight | Purgatives. Tobacco. Leeches. Mercury ... | —— xxiv. 277. |
| Fatal, following day | Opium. Mercury ......................... | —— xxx. 21. |
| Cured, a month .. | Purgatives. Opium. Mercury. Stimulants.. | —— xxx. 28. |
| Fatal, 12th day ... | Opium. Stimulants. Cantharides. Cold affusion. Mercury ................. | Med.&Phys.Jour. xiv. 129. |
| Fatal, 4th day.... | Warm-bath. Mercury .................. .. | —— xxi. 181. |
| Fatal, 2nd day.... | Mercury ...................................... | |
| Fatal, 4th day.... | ............................................ | —— xxi. 409. |
| Fatal, 3rd day.... | Blisters. Mercury............................ | —— xxi. 446. |
| Fatal ............ | Mercury ...................................... | —— |
| Fatal, 4th day.... | Blisters. Mercury.......................... .. | —— |
| Fatal, 4th day.... | Mercury. Amputation ..................... | —— xxii. 120. |
| Fatal, 5th day.... | Mercury ..... ............................... | —— |
| Cured, 7th day ... | Amputation.................................. | —— |
| Fatal, 2nd day.... | V.S. Opium. Mercury. Warm-bath ...... | —— xxii. 324. |
| Fatal, 5th day.... | Mercury. Division of wounded parts ....... .. | —— xxii. 185. |
| Cured, 8 weeks .. | Opium. Warm-bath. Stimulants. Mercury | —— xxii. 396. |
| Cured ........... | Mercury. Opium ........................... | —— xxii. 479. |
| Fatal, 2nd day.... | Mercury. Opium. Blisters ............... | Med. Quar. Rev. iv. |
| Cured ........... | Purg. Opium. Merc. Cold affus. V.S. Stimulants | Med. & Phys. Jour. xxxvii. 19. |
| Cured ........... | V.S. Purgatives. Opium ................. | xxxvii. 23. |
| Cured, 5 weeks ... | Opium. Warm-bath. Camphor. Hyoscyamus. V.S. Purgatives. Mercury | —— xliii. 192. |
| Cured, 6 weeks .. | Digitalis. Leeches. Antimony ............ | —— xliii. 289. |
| Cured, 4 days.... | V.S. Mercury. Purgatives. Warm-bath.. .. | —— xlvi. 538. |
| Cured, 5 weeks .. | Opium. Purgatives. Blisters. Hyoscyamus | —— l. 446. |
| Fatal, 2nd day.... | Purgatives. Hydrocyanic acid. V.S......... | —— lvi. 319. |
| Cured, a week.... | Purgatives. Leeches. Carbonate of iron. Mercury. Opium. Antim. Cold affusion | —— lxii. 220. |

| No. | Name. | Age. | Nature of the Injury. | Appearance of Symptoms. |
|---|---|---|---|---|
| 91 | Daniel Power | 40 | Fracture of the leg, with slight laceration of the integuments | 6th day |
| 92 | Patrick Vallily | 15 | Scalded on the leg, thighs, and arms, with hot water.... | 10th day |
| 93 | Wm. Fleming | 17 | ........................................ | 7th day |
| 94 | Thomas Lee.. | 45 | Compound fracture of three fingers .................. | 10th day |
| 95 | John Triggs.. | 25 | Slight wound on the outer ankle of the right leg ....... | 7th day |
| 96 | Samuel Grigg | 40 | A lacerated wound above the left elbow, and a scratch on the nose.............................. | 5th day |
| 97 | Pierre Genet . | 30 | A fall on the nose, causing excoriation and epistaxis .... | Five days |
| 98 | —Moustapha | 27 | Compound fracture, with laceration of the fingers and hand, from a gun-shot .................... | A month |
| 99 | M. Bonichon . | | Gun-shot wound of the left foot .................. | A fortnight |
| 100 | —— | 16 | A blow on the cervical spine.......................... | 3rd day |
| 101 | M. Leydet ... | | A compound fracture of the leg........................ | 7th day |
| 102 | —— | 28 | Last phalanx of auricular finger of left hand removed by a blow with a hatchet .................... | 2 hours |
| 103 | Edw. Wyer .. | | Sole of the left foot wounded with a nail.............. | 11th day |
| 104 | Joseph Parks | 15 | Fracture of the bones of the thumb with laceration .... | 6th day |
| 105 | Miles Bartley | 60 | A slight wound on the ear and temple..... ........... | 7 days |
| 106 | James Gaffney | 42 | Lacerated wound of the thumb........................ | 8 days |
| 107 | Mich. Farrell | 24 | A contusion and slight laceration on the right shin...... | 3 weeks |
| 108 | —— | 22 mo. | Fracture of the tibia and tarsus with laceration .... ... | 8th day |
| 109 | —— | 18 | Two fingers amputated on account of gun-shot wound .. | 10th day |
| 110 | —— | 38 | Fracture of both bones of left leg.................. | 23rd day |
| 111 | —— | 48 | Foot injured by a rusty nail ........................ | A week |
| 112 | —— | 40 | Fracture of the little finger ..... .................. | 9th day |

FEM[illegible]

| No. | Name. | Age. | Nature of the Injury. | Appearance of Symptoms. |
|---|---|---|---|---|
| 113 | M. Breaklock | 30 | A cut on the thumb .............................. | 4 days |
| 114 | Maria Ursin.. | 28 | Tendon of the peroneous longus exposed in an ulcer, pulled in dressing ......... .................. | An hour |
| 115 | —W....... | 40 | Lacerated wound on the palm of the left hand ........ | 11th day |
| 116 | A.B. a Negress | 40 | Cupping on the temples.............................. | 5 days |
| 117 | Acouba, a Neg. | mid. age | Incised wound of the hand and fingers................ | 13th day |
| 118 | —— | 50 | A compound fracture of the leg ..................... | 3 weeks |
| 119 | Elizabeth Fox | 23 | Lacerated wound of the ring finger of the right hand —last joint amputated ...................... | 16th day |
| 120 | E. E......... | 22 | Sole of the left foot wounded with a thorn ............ | 3 weeks |
| 121 | Ann Alford .. | 22 | Toes frost-bitten... ................................ | |
| 122 | Mrs. Martin.. | 52 | Wounded in the left cheek by an arrow .............. | Follows da[illegible] |
| 123 | Mary St.Gelais | | Laceration of the integuments of the right knee ........ | 18 days |
| 124 | Susan. a Neg. | 24 | Foot wounded with a bit of broken glass............... | 8th day |
| 125 | Mary A.Elliott | 14 | A lacerated wound of the wrist and palm of the hand.... | 14th day |
| 126 | —— | 37 | Compound fracture of the right leg .................. | 18th day |
| 127 | Justine, a Neg. | 18 | Amputation of the thigh ............................ | 10th day |
| 128 | ——, a Negress | 20 | A cut on the metatarsus ............................ | 10th day |

| Result, and in what time after. | TREATMENT. | AUTHORITY. |
|---|---|---|
| Fatal, following day | Mercury. Opium ................ ........ | Med. & Phys. Journ. lxvi. 42. |
| Fatal, 6th day.... | Purgatives. Opium. Mercury ............ | Glasgow Med. Jour. ii. |
| Fatal, 3rd day.... | Leeches. Mercury. Amputation. Antimony. Acetate of morphia ... ...... | —— ii. |
| Fatal, 26 hours .. | Purgatives. Mercury. Opium. Blisters .... | Medico-chirurg. Rev. x. 614. |
| Cured, 6 weeks ... | Dover's powders. Purgatives. Tonics ...... | Med. Transactions, iv. 29. |
| Fatal, 40 hours ... | .................................... | London Med. Repos. xiv. 1. |
| Fatal, 7th day.... | Opium. Warm-baths. Cautery............ | Larrey, Mémoires de Chirurgie Militaire, i. 241. |
| Cured, a month .. | Opium. Warm-baths. Camphor. Musk..... | —— i. 250. |
| Cured ........... | Amputation. Opium .................... | —— i. 265. |
| Fatal, same day .. | V.S. Purgatives ......................... | Dazille, Obs. sur le Tétanos. |
| Fatal, 3rd day.... | .................................... | —— |
| Fatal, 12 hours .. | V.S. Warm-bath. Opium................ | Dict. des Sciences Médicales. |
| Fatal, 9th day.... | Cold affusion. Tonics. Warm-bath. Electricity. Mercury .................. | Med. Facts and Obs. vii. 267. |
| Fatal, 2nd day.... | V.S. Purgatives. Opium. Cold affusion. Merc. | Transact. of the College of Physicians, Ireland, iv. 275. |
| Fatal, 6th day.... | Purgatives. Tobacco. Opium.............. | —— |
| Fatal, 3rd day.... | Tobacco. Mercury ....................... | —— |
| Cured, a fortnight | V.S. Purgatives. Mercury. Antimony. Stimulants. Opium ................ | —— |
| Fatal, 2nd day.... | .................................... | Travers on Const. Irrit. ii. 313 |
| Fatal, 3 days ..... | Purgatives. Belladonna ................. | —— |
| Fatal, 4th day.... | .................................... | —— |
| Fatal ........... | Belladonna ............... ........... | —— |
| Fatal, 48 hours ... | V.S.................................. | —— |

ALES.

| | | |
|---|---|---|
| Fatal, 19th day .. | Leeches. Opium. Antimony. Mercury. Zinc | Dr. Bright's Med. Rep. ii. 575 |
| Cured, 2 days .... | V.S. Leeches. Warm-bath. Mercury. Morphia | Medical Gazette, ii. 384. |
| Cured, a fortnight | Opium. Mercury. Turpentine ............ | —— vii. 428. |
| Cured ........... | Purgatives. Tobacco............. ....... | Edin. Med.-chir. Trans. i. 184. |
| Cured, 6 weeks .. | Purgatives. Tobacco............ ........ | —— |
| Cured, 5 weeks ... | Opium. Mercury ...................... | Med. chir. Transactions, ii. |
| Cured, a month .. | Blisters. Opium. Amputation ........... | Medical Obs. and Inq. i. 1. |
| Cured ........... | Blisters. Mercury. Opium. Cold affusion .. | Ed. Med. & Surg. Jour. i. 294. |
| Fatal, 2 days ..... | Opium. Mercury ........................ | Parry's Cases of Tetanus, 10. |
| Cured, a month .. | Opium. Purgatives. Mercury. Antimony .. | Med. & Phys. Jour. xxiv. 40. |
| Cured, a month .. | V.S. Purgatives. Opium. Mercury........ | —— xlii. 95. |
| Fatal, 5 days .. .. | V.S. Mercury. Opium. Stimulants, Warm-bath | Dr. Morrison's Treat. on Tetan. |
| Cured, 2 months.. | Purgatives. Opium. Tobacco ............ | Travers on Const. Irrit. ii. 323 |
| Fatal, 3rd day.... | Opium. Æther ......................... | —— ii. 316 |
| Cured, 3 months.. | Purgatives. Tobacco. Opium ....... ..... | Campet, Tr. des Mal. graves, 18 |
| Cured, 3 months.. | V.S. Purgatives. Opium. Tobacco........ | —— 22 |

